# Quantification and coupling of the electromagnetic and chemical contributions in surface-enhanced Raman scattering

**DOI:** 10.3762/bjnano.10.56

**Published:** 2019-02-25

**Authors:** Yarong Su, Yuanzhen Shi, Ping Wang, Jinglei Du, Markus B Raschke, Lin Pang

**Affiliations:** 1 College of Physical Science and Technology, Sichuan University, Chengdu, Sichuan 610065, China; 2 College of Physics and Electronic Engineering, Sichuan Normal University, Chengdu 610101, China; 3 Department of Physics, Department of Chemistry and JILA, University of Colorado at Boulder, Boulder, Colorado 80309, USA

**Keywords:** benzenethiol, chemical enhancement, physical enhancement, quantification, surface-enhanced Raman scattering (SERS)

## Abstract

In surface-enhanced Raman scattering (SERS), both chemical (CE) and electromagnetic (EM) field effects contribute to its overall enhancement. However, neither the quantification of their relative contributions nor the substrate dependence of the chemical effect have been well established. Moreover, there is to date no understanding of a possible coupling between both effects. Here we demonstrate how systematically engineered silver and gold planar and nanostructured substrates, covering a wide range of field enhancements, provide a way to determine relative contributions of chemical and electromagnetic field-enhancement in SERS measurements of benzenethiol. We find a chemical enhancement of 2 to 14 for different vibrational resonances when referencing against a vibrational mode that undergoes minimal CE. The values are independent of substrate type and independent of the enhancement of the electromagnetic intensity in the range from 1 to 10^6^. This absence of correlation between chemical and electromagnetic enhancement resolves several long-standing controversies on substrate and intensity dependence of the chemical enhancement and allows for a more systematic design of SERS substrates with desired properties.

## Introduction

Surface-enhanced Raman scattering (SERS) is a powerful spectroscopic technique for chemical analysis, providing molecular specificity through vibrational or rotational fingerprints. It has found widespread applications in surface science, materials research, and the life sciences [1–5]. Underlying the overall signal enhancement responsible for the exquisite sensitivity of SERS is a combination of both electromagnetic field effects and chemical effects [6–10]. It has been accepted that the dominant electromagnetic enhancement mechanism (EM) is the nanoscale field confinement associated with localized surface plasmon resonances of the nanostructured metal surface when excited by incident light. The generally weaker chemical enhancement mechanism (CE) is thought to be associated with electronic interactions such as charge redistribution, hybridization, or other interactions between molecular adsorbate and the metal substrate [11–12].

Depending on the degree of EM enhancement the signal simply scales linearly in intensity. In contrast, CE in addition can give rise to changes in the spectral response in terms of peak position and line shape due to modifications in molecular structure when the molecule chemisorbs on the metal surface [13]. This challenges the interpretation of spectroscopic signatures and provides difficulties for the development of SERS into a quantitative spectroscopy technique. Progress towards the quantitative distinction between CE and EM was only made recently under certain assumptions [14–17]. Further, a possible coupling between CE and EM was suggested. The plasmonic excitation field could reactively be affected by the excitation of molecules adsorbed on the metal substrate [18–19]. In addition, the vibrational motion of or within the adsorbed molecules could modulate the substrate polarizability, and thus enhance the Raman scattering [20–22]. This possible coupling effect was investigated theoretically, but not yet explored experimentally. Therefore, establishing a routine experimental procedure to separate and quantify CE and EM effects, as well as understanding the possible coupling between these two contributions would be essential to advance SERS into a routine analytical technique.

In this work, we demonstrate how SERS measurements of benzenethiol on a wide range of engineered metallic planar and nanostructured substrates, covering a large range of electromagnetic enhancement, can provide a way to separate and quantify CE and EM effects. Based on certain vibrational mode characteristics, the selection of a vibrational mode of only minimal CE, relative CE and EM values can be determined. Specifically, we take advantage of a vibrational mode characterized by a largely only intramolecular nuclear motion that is not simultaneously IR active. This has been suggested to give rise to an only minimal change in deformation potential, i.e., a negligible change in the molecular frontier orbital energy [14–17]. Choosing such a vibrational mode as an internal reference then allows for the separation of EM and CE, and the quantification of CE of other modes referenced against that standard. Without loss of generality, and setting the reference CE value to unity, we find a vibrational-mode-specific relative chemical enhancement ranging from 2 to as large as 14 for different vibrational modes studied, yet with no dependence on the kind of metal substrate. Further, for the electromagnetic enhancement ranging from 1 to 10^6^ for the different substrates used we find no correlation between CE and EM effects. We expect this constant chemical enhancement and independence between chemical and electromagnetic enhancement to allow for a more systematic design of SERS substrates with desired properties to turn SERS into a quantitative analytical technique.

## Experimental

**Figure 1:**
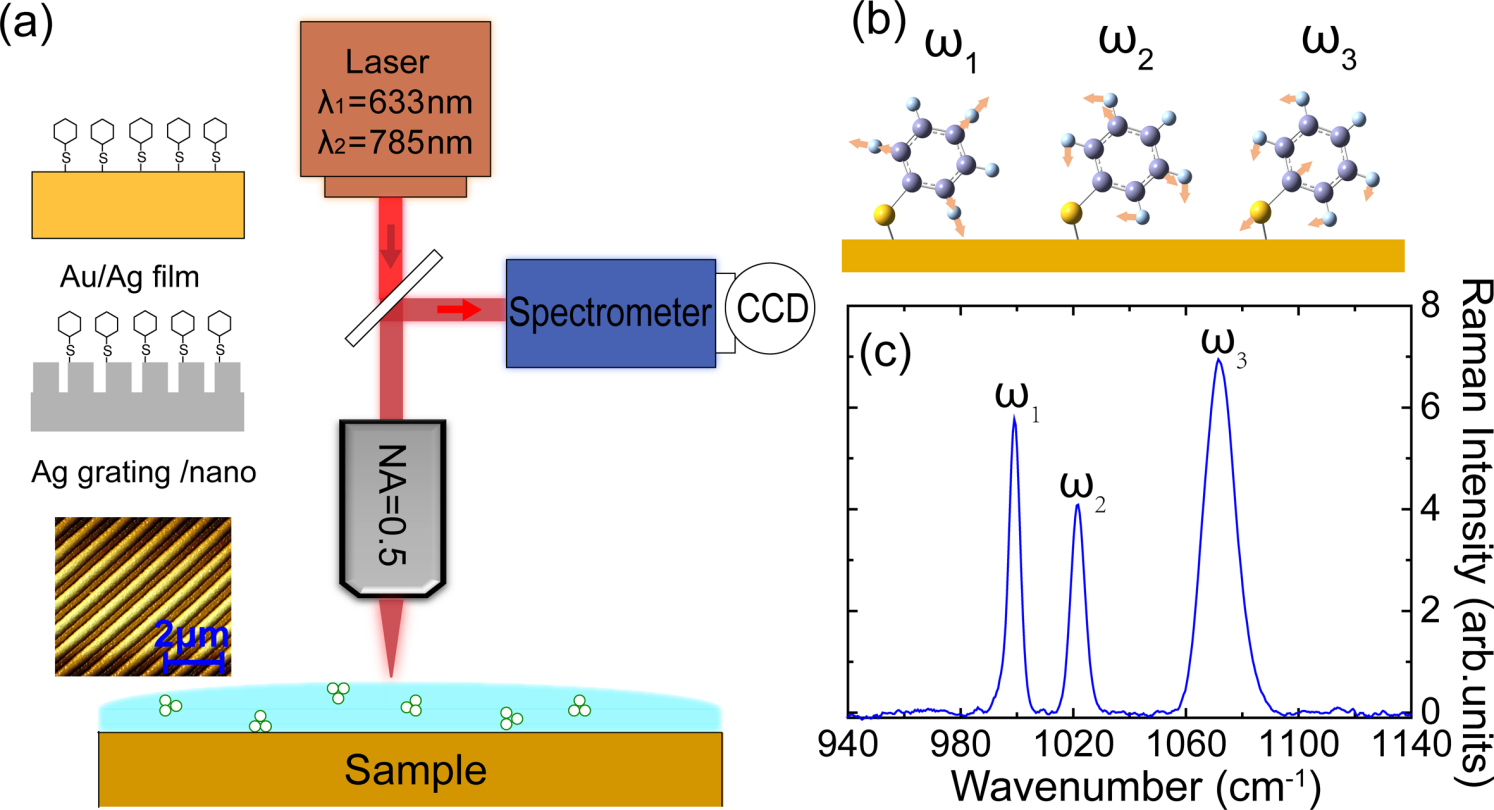
(a) Schematic of experimental arrangement and range of SERS substrates used with different enhancements. (b) Vibrational resonances of benzenethiol with nuclear motion as indicated and (c) corresponding SERS spectrum on a Ag nanosubstrate.

A schematic of the experimental approach is shown in Figure 1. Different Au and Ag metal substrates were prepared and chemically functionalized with benzenethiol. Planar Au and Ag films were prepared by metal evaporation, with minimal grain texture and boundaries providing for the smallest EM enhancement of the samples investigated. Nanostructured Ag substrates were fabricated by interference lithography (IL) and oblique angle deposition (OAD) methods [23]. The Ag substrates with nanostructures are gratings with and without nanogap, which can achieve field enhancements as high as 10^6^. The samples were pre-characterized by AFM (see example of Ag grating with nanogap in inset in Figure 1a, for details see Supporting Information File 1). The metal substrates were first submerged in 1 mM benzenethiol solution in ethanol for 2 h and then gently rinsed in ethanol for 1 min, followed by drying in nitrogen flow [24].

Raman spectroscopy was performed using a standard Raman microscope (HORIBA LabRAM HR Evolution, with a 50×, NA = 0.5, long working distance objective, spectral resolution 0.5 cm^−1^) with 633 and 785 nm laser excitation with beam sizes of 1.5 and 1.9 μm, and laser powers of 2.6 and 3.8 mW, respectively, unless indicated otherwise. The Raman spectra of liquid benzenethiol were measured as a reference to estimate the SERS enhancement factor. All spectra were recorded with 5 s acquisition time. The silicon phonon Raman response at 520.7 cm^−1^ was used to calibrate the spectrometer.

## Results

### SERS spectra

The spectral range of 900–1200 cm^−1^ was selected for the study of three characteristic Raman modes of benzenethiol at 1000 cm^−1^ (ω_1_), 1025 cm^−1^ (ω_2_), and 1092 cm^−1^ (ω_3_). As shown schematically in Figure 1b, the ω_1_ mode is an out-of-plane ring deformation, ω_2_ modes are the in-plane ring deformation and C–C symmetric stretch, and ω_3_ modes are C–C asymmetric stretch and C–S stretch modes [17]. Only the ω_3_ mode is thought to be sensitive to charge redistribution due to the nuclear motion of the S-atom with respect to metal substrate and phenyl ring [15]. DFT calculations (Gaussian 09 package) were performed based on benzenethiol bound to three silver atom clusters, for illustration, and the assignment results of the vibrational modes are in agreement with [25].

The ω_1_ mode is not IR active and only a change in deformation potential would contribute to its possible CE as proposed in [15]. Its out-of-plane ring mode is characterized by a largely only intramolecular motion, minimizing modulation of the electronic deformation potential regarding the alignment of the HOMO with respect to the Fermi energy. As shown in [15] this gives rise to an only small CE, possibly close to unity, and makes this mode a suitable candidate as an internal reference standard for the quantification of CE of other Raman active modes of benzenethiol on different substrates and when limited to within a fraction of the localized surface plasmon bandwidth.

**Figure 2:**
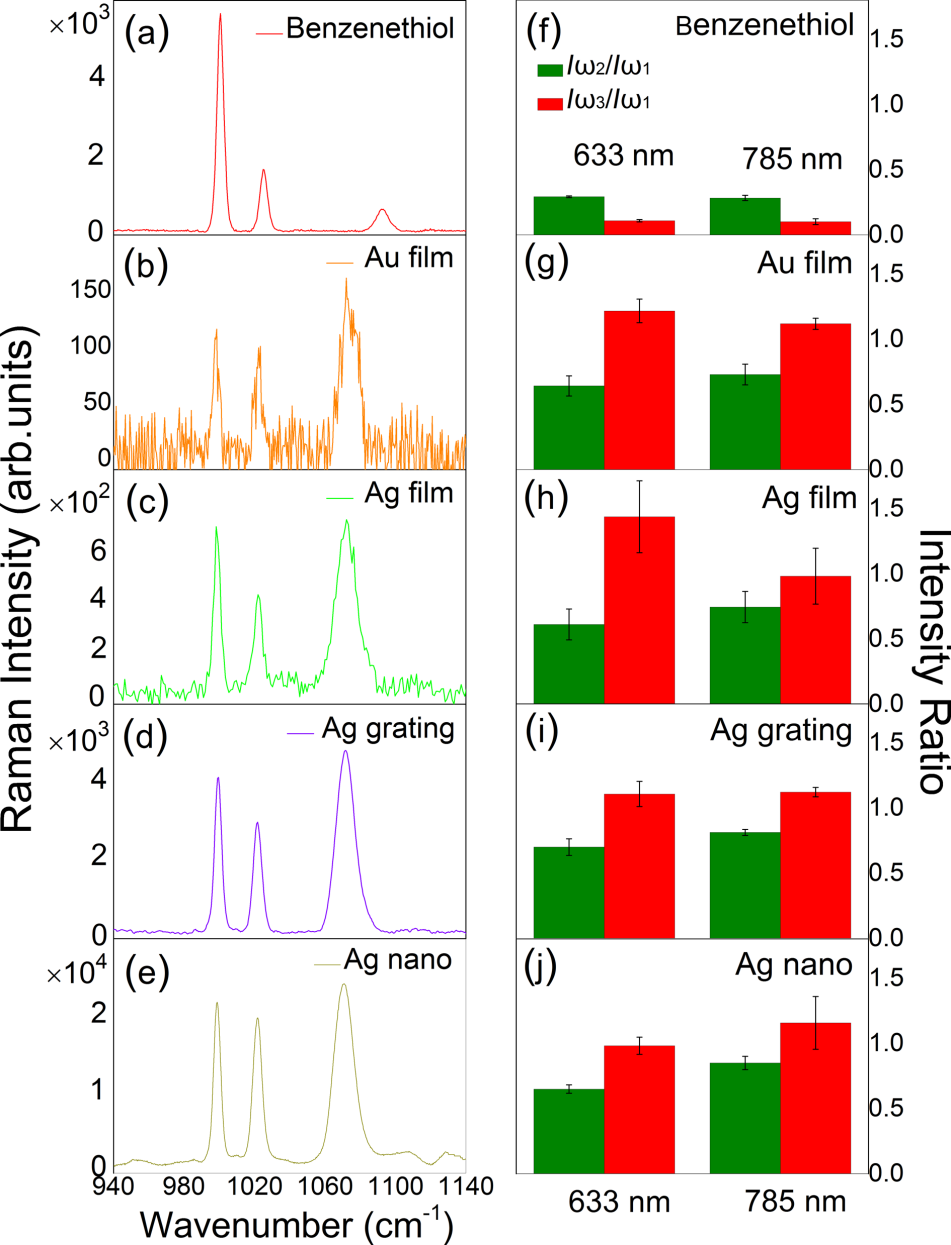
Raman spectra of benzenethiol on different substrates for 633 nm excitation (corresponding data for 785 nm excitation, see Supporting Information File 1). (a) Liquid benzenthiol, (b) Au film, (c) Ag film, (d) Ag grating, and (e) Ag nanostructure. (f–j) Corresponding intensity ratios of the ω_2_ and ω_3_ modes normalized to the ω_1_ modes for both 633 nm and 785 nm excitation. Each data set is the average from measurements at ten different sample locations. The error bars represent the standard deviations.

Representative Raman spectra of self-assembled monolayers of benzenethiol acquired on four different metal substrates, in comparison to neat benzenethiol are displayed in Figure 2a–e. A significant change in the intensity ratio of the different modes is seen, as compared to the neat benzenethiol spectrum. The observed dominance of the ω_3_ mode is the result of the charge redistribution from the Au/Ag surface clusters to the S atom in benzenethiol associated with its nuclear motion.

From a qualitative inspection it is already evident that the relative intensity ratios *I*(ω_2_)/*I*(ω_1_) and *I*(ω_3_)/*I*(ω_1_) are largely unaffected by the type of substrate and irrespective of the overall SERS enhancement. For a detailed quantitative comparison, we normalize the intensities with respect to the intensity of ω_1_. On the Au and Ag film substrates, we find intensity ratios of *I*(ω_2_)/(Iω_1_) = 0.64–0.745 and *I*(ω_3_)/*I*(ω_1_) = 0.98–1.44, with details and range depending slightly on excitation wavelength and type of substrate as discussed below. However, as seen in Figure 2f–j, as we increase the local field enhancement in the transition from planar to nanostructured substrates of variable SERS enhancement, the intensity ratios of *I*(ω_2_)/*I*(ω_1_) and *I*(ω_3_)/*I*(ω_1_) remain largely constant within the uncertainty of the experiment.

### Enhancement factor calculation

To estimate the underlying CE and EM enhancement factors, the Raman spectrum of neat benzenethiol is used as reference. All measurements were normalized to account for differences in surface coverage, laser power, and acquisition time, before enhancement factor calculation (for details see Supporting Information File 1).

The Raman enhancement factor (EF) is calculated using

[1] $$\text{EF}=\left(\frac{I_{\text{SERS}}}{I_{\text{Raman}}}\right)\cdot \left(\frac{N_{\text{Raman}}}{N_{\text{SERS}}}\right),$$

where *I*_SERS_ is the integrated Raman intensity of a surface-enhanced Raman mode for benzenethiol adsorbed on a given substrate, and *I*_Raman_ is the intensity of the same Raman mode for neat benzenethiol; *N*_Raman_ and *N*_SERS_ are the number of molecules contributing to the neat Raman signals and SERS signals of benzenethiol, respectively. *N*_Raman_ is given by

[2] $$N_{\text{Raman}}=\rho \cdot V\cdot \frac{N_{\text{A}}}{\text{ME}},$$

where ρ is the density (1.073 g/mL) of benzenethiol in the neat liquid, *V* is the optical excitation volume, *N*_A_ is Avogadro’s number, and ME (110.18 g/mol) is the molecular weight of benzenethiol. *N*_SERS_ is obtained from

[3] $$N_{\text{SERS}}=\rho_{\text{surf}}\cdot N_{\text{A}}\cdot S_{\text{surf}},$$

where ρ_surf_ (0.544 nmol/cm^2^) is the surface coverage of benzenethiol [24], and *S*_surf_ is the size of the laser spot.

The results are summarized in Table 1 for the enhancement factors for the three vibrational modes investigated and the four different metal substrates with 633 nm excitation (for 785 nm excitation see Table S1 in Supporting Information File 1). The enhancement factor values for ω_1_ = 1000 cm^−1^ are as low as 7.8 for benzenethiol adsorbed on the mostly smooth Au film, 3.1 × 10^2^ on the Ag film, reaching 1.3 × 10^4^ on the Ag grating, and as high as 1.2 × 10^6^ on the granular Ag nanosubstrate. Due to the spatial heterogeneity of the substrates, the enhancement factors are found to vary by about one order of magnitude within the same sample. Measurements were repeatable for individual sample locations within statistical errors. For each kind of substrate, three or more samples were measured. For each sample, different sample locations were measured, compared, and averaged as needed.

**Table 1:** Raman enhancement factors for different substrates with 633 nm excitation.

substrate	EF			EFω_3_/EFω_1_	EFω_2_/EFω_1_
	ω_1_	ω_2_	ω_3_		

Au film	7.8	24	1.1 × 10^2^	12 ± 1	2.1 ± 0.1
Ag film	3.1 × 10^2^	5.8 × 10^2^	3.9 × 10^3^	13 ± 2	2.1 ± 0.1
Ag grating	1.3 × 10^4^	3.1 × 10^4^	1.4 × 10^5^	11 ± 1	2.4 ± 0.5
Ag nano	1.2 × 10^6^	2.7 × 10^6^	1.2 × 10^7^	12 ± 2	2.5 ± 0.3

The Raman tensor is related to the square of energy difference between Fermi level of the metal and HOMO of molecule ω_H_ = *E*_F_ − *E*_HOMO_, and the corresponding deformation potential ∂ω_H_/∂Q_n_ [15]. With ω_H_^2^ equal for all modes, thus not responsible for the mode-dependence of CE the deformation potential ∂ω_H_/∂Q_n_ can reflect the mode-dependence of CE, through the change in molecular electronic level alignment, relative to *E*_F_, induced by a particular vibrational motion. The value for the chemical enhancement can be obtained by normalization to a mode with zero deformation potential ∂ω_H_/∂Q_n_. As for the ω_1_ = 1000 cm^−1^ mode, it was proposed that there is almost no change of the frontier energy levels as the atoms vibrate, and very little interfacial contribution to the change in polarizability [15]. Thus, only the electromagnetic enhancement has been thought to contribute to the SERS enhancement of the ω_1_ mode to a good approximation. We therefore use this mode as reference to calculate the chemical enhancement for the nearest two modes ω_2_ = 1025 cm^−1^, and ω_3_ = 1092 cm^−1^. Within the narrow spectral range of Raman modes studied, we can neglect spectral variations of the electromagnetic field enhancement, e.g., due to plasmonic effects, and assume that the electromagnetic enhancement is the same for all modes. The relative enhancement will then reflect the variations of CE for the different modes. We find that the relative enhancement of EF(ω_3_)/EF(ω_1_), which represents the CE contribution to ω_3_, varies between 9.8 and 14.3. Correspondingly, the relative CE enhancement value of EF(ω_2_)/EF(ω_1_) is 0.16–2.5. The corresponding spectral shifts Δω_i_ for the three modes for surface-adsorbed benzenethiol compared to liquid benzenethiol under 633 nm excitation correlate with the degree of CE with shifts of only ≈1 cm^−1^ ω_1_ in contrast to ≈3 cm^−1^ for ω_2_ and 18–21cm^−1^ for ω_3_ (for details see Supporting Information File 1, Tables S2–S5). The spectral shifts are independent of the type of substrate and similar for 785 nm excitation (see Supporting Information File 1).

**Figure 3:**
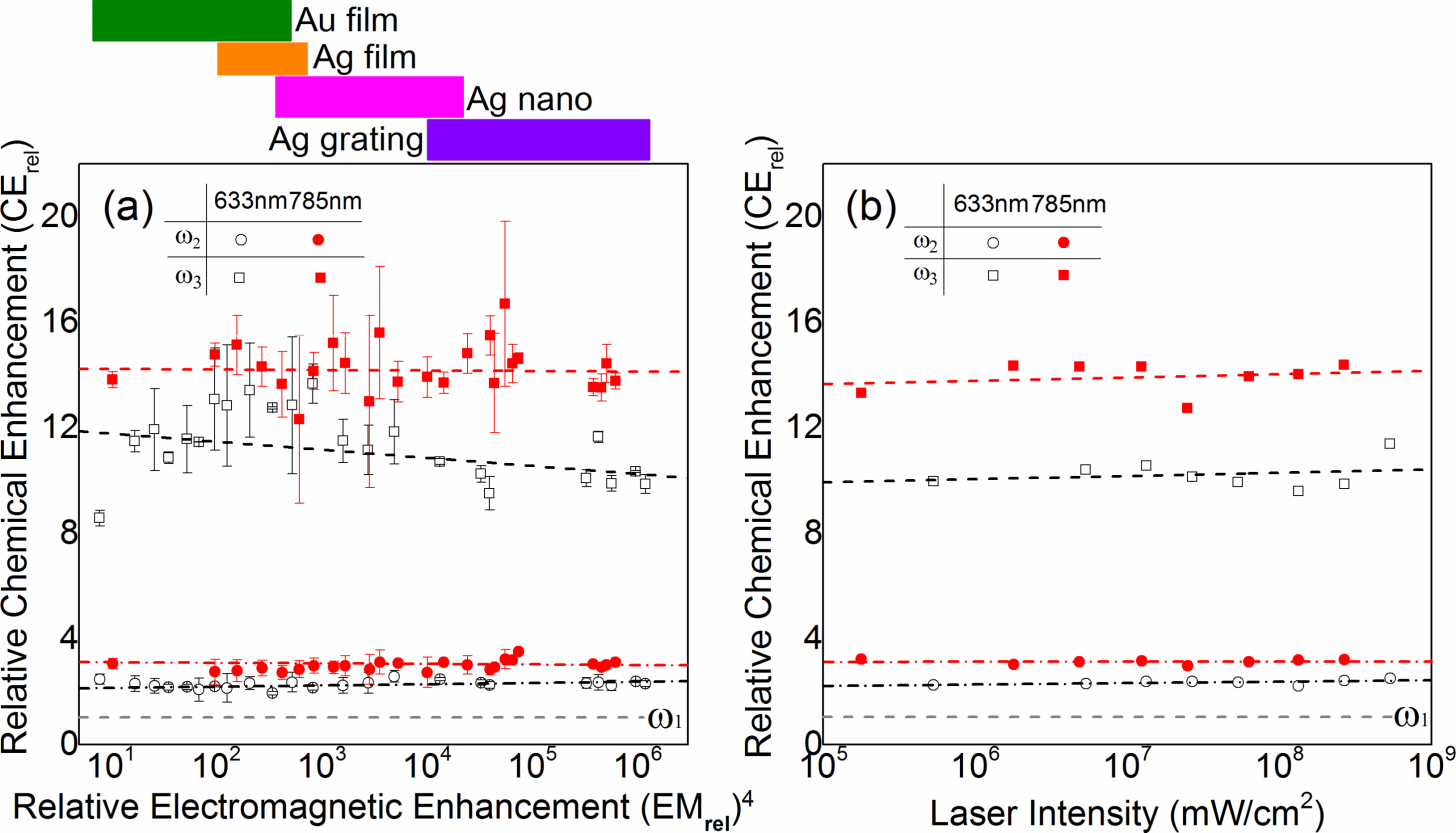
(a) Relative Chemical enhancement (CE_rel_) factor versus relative electromagnetic enhancement (EM_rel_) for different substrates with 633 nm (black) and 785 nm (red) excitation (with incident laser power of 2 × 10^7^ mW/cm^2^) ω_2_ and ω_3_. (b) Chemical enhancement as a function of incident laser intensity for the Ag nanosubstrate with 633 nm (black) and 785 nm (red) excitation.

The results for CE as a function of (EM)^4^ are plotted in Figure 3a for the range of samples studied and their corresponding range of EM enhancement. The dashed lines are linear fits for the mode ω_3_, to the data with CE = (−0.02 ± 0.07) × (EM)^4^ + (14 ± 0.32) for 785 nm and CE = (−0.31 ± 0.05) × (EM)^4^ + (12 ± 0.15) for 633 nm, respectively, within the EM enhancement range of 10 to 10^6^. Similar fits to ω_2_ yield CE = (−0.02 ± 0.03) × (EM)^4^ + (3.1± 0.15) for 785 nm, and CE = (0.05 ± 0.01) × (EM)^4^ + (2.1 ± 0.04) for 633 nm, respectively. Although a statistically significant slight decrease in CE with increasing EM is seen for 633 nm excitation, within the overall systematic uncertainty of the experiment, we judge that for both modes the chemical enhancement shows no systematic dependence on the kind of metal substrate and EM value.

Similarly, we then performed SERS measurements as a function of laser power on representative granular Ag nanosubstrates with results shown in Figure 3b. Just like the variation of EM values within and between different samples, the variation in excitation fluence models variations in local field enhancement (EM). Correspondingly, we observe similar CE values, independent of excitation fluence, within the range of powers investigated, limited by the signal-to-noise-ratio for low powers, and thermal effects at high powers. The dashed lines are linear fits to the data with CE = 1.9 × 10^−9^ × (Power)^4^ + 13 for 785 nm and CE = 1.7 × 10^−9^ × (Power)^4^ + 10 for 633 nm, respectively, within the incident laser power range of 10^5^ to 10^9^ mW/cm^2^.

Interestingly, despite the differences in absolute magnitude of chemical enhancement for both modes their relative change with laser wavelength is similar. From the average of the aggregate of the data, the relative change CE(633nm)/CE(785nm) is 0.76 ± 0.05 for ω_2_ and 0.79 ± 0.11 for ω_3_. Similarly, from the laser intensity dependent measurements, the corresponding relative change CE(633nm)/CE(785nm) is 0.84 ± 0.04 for ω_2_ and 0.75 ± 0.04 for ω_3_.

## Discussion

In the following we discuss the origin and implication of the observation that even for the electromagnetic enhancement ranging from as low as 10 to as high as 10^6^ for the different substrates used, we find no obvious correlation between CE and EM effects. However, the CE varies with excitation frequency, which indicates that CE is frequency-dependent but not particularly sensitive to the strength of the local optical field within the range of frequencies and field strengths studied. The CE corresponds to the static chemical enhancement (CHEM), which is induced by non-resonant changes in the molecular polarizability upon adsorption on the metal surface [17]. This interface contribution is correlated (to the first order) with the deformation potential and the enhancement of most vibrational modes [15,26]. Therefore, it is believed that the vibrational modes that exhibit the largest interfacial contribution to the modification in polarizability, as expressed by means of the deformation potential, exhibit the largest amount of chemical enhancement.

The ω_1_ mode is considered to fulfil this attribute as a reference mode, with minimal CE enhancement itself, and its small to negligible spectral shift upon surface absorption supports this assumption. By taking advantage of the dependence of the chemical enhancement on the vibrational mode, the dominant contributions to CE can then be determined quantitatively. The theoretical CE values range from 9 to 16 for ω_3_ depending on model details, but are overall in good agreement with our experimental value of ≈14, which further agrees with a value of 12 obtained from an earlier theory of small cluster models of a metal–benzenethiol complex excited at 785 nm wavelength [16]. Our values further agree well with experimental CE values of ≈11 for 785 nm and 8 ± 2 for 633 nm obtained on a rough gold surface [10], including the trend to higher values for longer wavelengths. Computed excitation profiles for chemical enhancement in [16], however, showed the chemical enhancement to decrease with decreasing photon energy. However, the exact experimental conditions are not captured in that theory, and the spectral variations are found to be sensitive to the electronic structures of the metal–molecule hybrid state. The calculated electronic excitation spectra of different complexes are quite dissimilar, which is related to the relative orientation of the benzene ring with respect to the cluster, the local symmetry of the benzene ring, and the proximity of the particular vibrational mode to the binding site [27].

A single, unified expression for SERS was proposed, including various resonant contributions based on Herzberg–Teller vibronic coupling terms to deduce the relationship between resonances [28–29]. This suggests that across a charge-transfer or molecular resonance, the enhancement of the asymmetric vs symmetric mode varies. Yet, in the absence of charge-transfer contributions, all modes are equally enhanced, irrespective of excitation frequency [29]. In our work, the mode ω_3_ is related with the charge-transfer contribution, and its CE value depends on the excitation wavelength.

With regards to coupling between physical and chemical enhancement [20,22], a coupling factor can be introduced to quantify how the induced polarization modifies the charge redistribution and vice versa. However, we observe no such coupling in our experimental results, as the chemical enhancement remains almost constant within a large variation of physical enhancement.

Physical and chemical enhancement both contribute to the changes in the spectra. The static chemical contribution is due to an increase of the polarizability derivative, which in turn is due to a change in the ground-state electronic structure when the molecule adsorbs onto a metal surface. In addition, dynamic processes contribute, which are associated with the formation of hybrid states or charge transfer excitations between the molecule and the metal [16]. Chemical enhancement is frequency dependent through vibrational coupling, thus the Raman response from the metal–molecule complex is resonantly enhanced even at energies below an actual molecular electronic excitation [16,22,29]. A high value of CE may be obtained at a resonant frequency because the electronic structure of a metal–molecule system is very sensitive to the increase of the excitation photon energy [29].

In addition to the excitation energy, the chemical mechanism in SERS is sensitive to the local molecular environment and the property of the metal surface. As for the molecule–metal complex, which includes the relative orientation of the molecule with respect to the metal cluster, the local symmetry of molecule, and the proximity of the particular vibrational mode to the binding site all contribute to SERS. Besides, specific properties of the Raman response are strongly dependent on the local atomic environment of the adsorbate. Optical properties, such as the locations of the optical transitions, oscillator strengths, and homogeneous linewidths, are also critical for molecule–metal complex systems [29]. With improved knowledge of the resonance characteristics and oscillator strengths, the mechanism of various contributions to the SERS intensities could be obtained, which would provide new opportunities in quantitative detection through SERS.

Overall, the absence of any detectable correlation between CE and EM suggests that charge redistribution might not contribute significantly to CE in this case. Additionally, an increase in the local optical field strength, whether through an increase in field enhancement or laser intensity, would give rise to an increase in excitation density of electrons at the Fermi level and thus effect the electronic distribution. Instead it may be the strong sulfur–metal bond itself with its effect on the electronic structure of the phenyl group that leads to the modification and CE of the Raman polarizability of the associated modes. Similarly, a direct modification of the magnitude of CE through a vibrational Stark effect is not expected. Even for highest enhancement and laser intensity, the local optical field is only of the order of 10^5^ V/cm, which is still significantly lower compared to both intermolecular electrostatic fields (10^6^–10^7^ V/cm) and intramolecular electrostatic fields (10^7^–10^8^ V/cm) [30]. The interpretation above is based on the assumptions that the out-of-plane ring deformation for ω_1_ at 1000 cm^−1^ exhibits a CE of unity value, and that the EM is the same for all the three modes [15]. Possible future and more advanced theories might refine this picture, yet can directly relate to our experimental ratios by simply providing a correction factor to obtain updated absolute values of CE. The experimental results and the independency of CE and EM still hold and only their relative values would change. The observed results are certainly specific to the employed analyte benzenethiol.

## Conclusion

This work demonstrates a systematic approach based on engineered substrates covering a wide range of enhancement values to gain access to the relative contributions of chemical and electromagnetic enhancement in SERS. It allows one to extract and distinguish values of the relative chemical enhancement, ranging from 2 to 14, for different vibrational resonances in benzenethiol. Irrespective of the absolute values, they are independent of the substrate type and independent of the electromagnetic intensity enhancement in the range from 1 to 10^6^. The observed independence of the chemical enhancement from the physical enhancement provides for a novel design principle to optimize SERS substrates for sensing and photocatalysis in a new systematic manner for quantitative analysis and photochemistry. New theoretical work to investigate the underlying electronic and vibronic structure of the metal–molecule system under different excitation photon energies is desirable. This work points the way towards its extension to higher excitation fields (transient and static) to explore the expected CE–EM coupling and nonlinear SERS regimes, and towards a dynamic non-perturbative SERS response.

## Supporting Information

Additional information on the structure of the substrates used (Figure S1), the corresponding SERS spectra for 785 nm excitation (Figure S2), with tables of Raman enhancement factors under 785 nm excitation (Table S1), spectral shifts for 633 nm (Table S2) and 785 nm (Table S3) excitation, and the ratios of spectral shift to relative chemical enhancement under 633 nm (Table S4) and 785 nm (Table S5) excitation.

File 1 Additional experimental data.
