# Supplementary material for: Quantification and coupling of the electromagnetic and chemical contributions in surface-enhanced Raman scattering
Source: Beilstein J Nanotechnol. 2019 Feb 25;10:549–56. doi: 10.3762/bjnano.10.56 (PMC6404390; doi:10.3762/bjnano.10.56)
Supplement: File 1 — Additional experimental data. [file Beilstein_J_Nanotechnol-10-549-s001.pdf]

BEILSTEIN JOURNAL OF NANOTECHNOLOGY

Supporting Information

for

Quantification and coupling of the electromagnetic and chemical contributions in surface-enhanced Raman scattering

Yarong Su, Yuanzhen Shi, Ping Wang, Jinglei Du, Markus B. Raschke and Lin Pang

Beilstein J. Nanotechnol. **2019,** *10,* 549–556. doi:10.3762/bjnano.10.56

Additional experimental data

License and Terms: This is a supporting information file under the terms of the Creative Commons Attribution License (http://creativecommons.org/licenses/by/4.0). Please note that the reuse, redistribution and reproduction in particular requires that the authors and source are credited.
The license is subject to the *Beilstein Journal of Nanotechnology* terms and conditions: (https://www.beilstein-journals.org/bjnano)

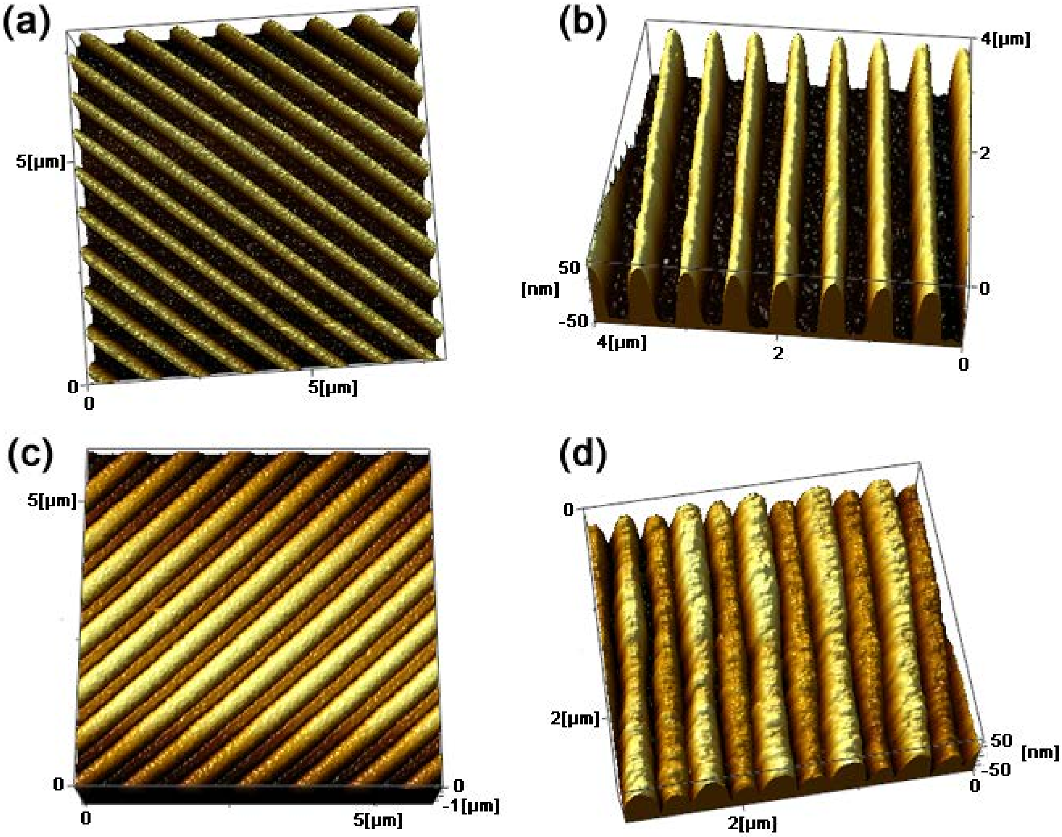

Figure S1: AFM images of (a, b) Ag grating substrate and (c, d) Ag nanosubstrate.

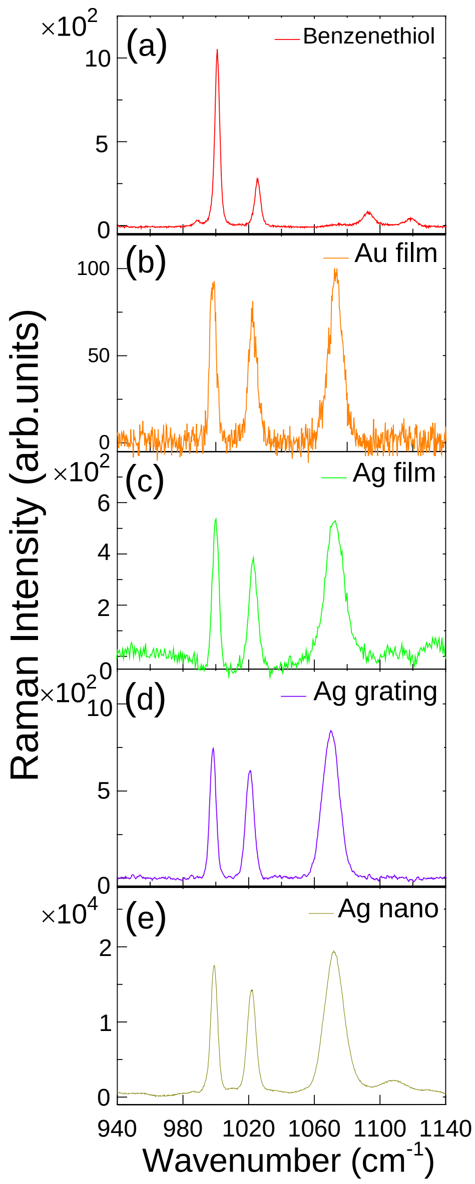

Figure S2: Raman spectra of (a) neat benzenethiol and benzenethiol adsorbed on different kinds of substrates under 785 nm excitation: (b) planar Au film, (c) planar Ag film, (d) Ag grating, and (e) Ag nanostructures.

Normalization of Raman intensity

I_{SERS} is the integrated Raman intensity of a surface-enhanced Raman mode for benzenethiol adsorbed on a given substrate:

$$I_{normalized} = \frac{I_{SERS}}{I_{Neat}} \cdot \frac{P_{Neat}}{P_{SERS}} \cdot \frac{t_{Neat}}{t_{SERS}}$$

with I_{Neat} the intensity of the same Raman mode for the liquid benzenethiol. P_{Neat} and P_{SERS} are the respective excitation powers used ,and t_{Neat} and t_{SERS} correspond to integration times in the measurements.

Table S1: Raman enhancement factors for different substrates with 785nm excitation.

Substrate	EF			$\frac{EF\omega_3}{EF\omega_1}$	$\frac{EF\omega_2}{EF\omega_1}$
	ω_1	ω_2	ω_3		
Au film	82	2.8×10^2	1.2×10^3	15 ± 1	2.7 ± 0.2
Ag film	9.1×10^2	2.8×10^3	1.26×10^4	13 ± 2	2.7 ± 0.3
Ag grating	5.5×10^4	1.8×10^4	8.1×10^4	14 ± 1	3.1 ± 0.1
Ag Nano	1.2×10^6	2.7×10^6	1.2×10^7	16 ± 3	3.2 ± 0.5

Table S2: Spectral shifts of modes ω_1, ω_2, and ω_3 for surface adsorbed vs liquid benzenethiol on the four different substrates. The frequencies are given in cm^{-1}. The excitation wavelength is 633 nm.

Substrate	ω_1	$\Delta\omega_1$	ω_2	$\Delta\omega_2$	ω_3	$\Delta\omega_3$
Liquid	1000	—	1025	—	1092	—
Au film	999	−1	1022	−3	1074	−18
Ag film	998	−2	1022	−3	1072	−20
Ag grating	999	−1	1022	−3	1072	−20
Ag Nano	999	−1	1022	−3	1071	−21

Table S3: Spectral shifts of modes ω_1, ω_2, and ω_3 for surface adsorbed vs liquid benzenethiol on the four different substrates. The frequencies are given in cm^{-1}. The excitation wavelength is 785 nm.

Substrate	ω_1	$\Delta\omega_1$	ω_2	$\Delta\omega_2$	ω_3	$\Delta\omega_3$
Liquid	1000	—	1025	—	1093	—
Au film	999	−1	1022	−3	1074	−19
Ag film	998	−2	1022	−3	1073	−20
Ag grating	999	−1	1021	−4	1071	−22
Ag Nano	999	−1	1022	−3	1070	−23

Table S4: The ratios of spectral shift to chemical enhancement. The excitation wavelength is 633 nm.

Substrate	$\Delta\omega_2/\Delta\omega_1$	$CE\omega_2$	$\frac{\Delta\omega_2/\Delta\omega_1}{CE\omega_2}$	$\Delta\omega_3/\Delta\omega_1$	$CE\omega_3$	$\frac{\Delta\omega_3/\Delta\omega_1}{CE\omega_3}$
Au film	3	2.1 ± 0.1	1.43	18	12 ± 1	1.5
Ag film	1.5	2.1 ± 0.1	0.71	10	13 ± 2	0.77
Ag grating	3	2.4 ± 0.5	1.25	20	11 ± 1	1.82
Ag Nano	3	2.5 ± 0.3	1.2	21	12 ± 2	1.75

Table S5: The ratios of spectral shift to relative chemical enhancement. The excitation wavelength is 785 nm.

Substrate	$\Delta\omega_2/\Delta\omega_1$	$CE\omega_2$	$\frac{\Delta\omega_2/\Delta\omega_1}{CE\omega_2}$	$\Delta\omega_3/\Delta\omega_1$	$CE\omega_3$	$\frac{\Delta\omega_3/\Delta\omega_1}{CE\omega_3}$
Au film	3	2.7±0.2	1.11	19	15±1	1.27
Ag film	1.5	2.7±0.3	0.56	10	13±2	0.77
Ag grating	4	3.1±0.1	1.29	22	14±1	1.57
Ag Nano	3	3.2±0.5	0.94	21	16±3	1.31
